# Cytological endometritis and its agreement with ultrasound examination in postpartum beef cows

**DOI:** 10.14202/vetworld.2017.605-609

**Published:** 2017-06-07

**Authors:** N. Salah, N. Yimer

**Affiliations:** 1 Department of Clinical Studies, Faculty of Veterinary Medicine, Universiti Putra Malaysia, UPM 43400 Serdang, Selangor, Malaysia; 2 Department of Obstetrics and Surgery, Faculty of Veterinary Medicine, University of Diyala, Baquba, 00964, Iraq

**Keywords:** beef cows, cytology, endometritis, polymorphonuclear cells, ultrasound

## Abstract

**Background::**

Endometritis, which is one of the most common diseases in dairy cows postpartum, causes severe economic losses, including increased open days, calving intervals, and numbers of services to achieve conception.

**Aim::**

This study aimed to evaluate the ultrasound method and its agreement with the endometrium cytology method, which is used to diagnose cytological endometritis in beef cows. Moreover, we determined which method has higher sensitivity and specificity at 4 and 5 weeks postpartum.

**Materials and Methods::**

The study was conducted 20-35 days postpartum. A total of 53 clinically healthy beef cows (28 Brangus and 25 Kedah–Kelantan breeds) from three beef farms were obtained. All cows were evaluated at 4 and 5 weeks postpartum, using ultrasound and cytobrush endometrial examination methods to diagnose cytological endometritis.

**Results::**

Endometrial cytology result showed that 11.3% (6/53) and 9.4% (5/53) of the cows exhibited cytological endometritis 4 and 5 weeks postpartum, respectively. A weak-to-moderate agreement found between the diagnostic methods (k=0.29 - 0.50; p<0.01 and k=0.38 - 0.49) at 4 and 5 weeks postpartum respectively.

**Conclusion::**

The percentage of beef cows that were positive to cytological endometritis was low (polymorphonuclear cells, ≥8%) at 4 and 5 weeks postpartum. Results showed that the ultrasound method is useful and practical for diagnosing endometritis 4 and 5 weeks postpartum. This method exhibited 60% sensitivity, 93.8% specificity, and a 0.50 kappa value, especially when presence of intrauterine fluids and measurement of cervix diameter used in combination.

## Introduction

The uterus is exposed to several types of microbial contamination after parturition that can cause severe economic losses to farmers due to abortions, infertility, and death. Uterine infections can be classified as puerperal metritis, clinical metritis, clinical endometritis (CE), and subclinical endometritis (SCE). SCE is the inflammation of the uterine endometrium without mucopurulent material accumulation in the vagina and any systemic symptom [1]. SCE is also known as cytological endometritis [2,3]. Dubuc *et al*. [3] described cytological endometritis as “an elevated ratio of polymorphonuclear cells (PMN) in endometrial cytology samples obtained through cytobrush (CB) or low-volume uterine lavage (LVF).” CE is an endometrial inflammation with purulent or mucopurulent discharge; moreover, this disease can be detected 21 days postpartum and is associated with clinical signs of disease [1]. The term “purulent vaginal discharge” was adopted as a substitute for CE because the presence of abnormal genital discharge does not necessarily indicate endometrial inflammation [3]. Endometritis is prevalent in highly productive dairy cows and has been associated with decreased pregnancy per insemination, extended pregnancy intervals, and increased culling rate [2].

Precise diagnosis of endometrial infections in cows is hindered by the lack of consensus on an acceptable definition of bovine endometritis [1,2]. Most cows experience some degree of endometritis during normal uterine involution after birth. Transrectal palpation of the uterus is the most common method of diagnosing postpartum uterine diseases; however, this method lacks the accuracy to identify endometritis and subsequent reduced fertility [4,5]. Several approaches, such as the collection of endometrial and inflammatory cells using a guarded cotton swab [6], uterine biopsy [7], LVF [2], or CB [8], are used to detect cytological endometritis. Moreover, CB and LVF are less invasive techniques compared with uterine biopsy [9]. The CB method is less harmful than LVF because the fluid (normal saline, 0.9%) used in LVF produces endometrial irritation. Moreover, the saline solution extends the time required to obtain samples (a 17% failure to obtain saline) and increases the alteration of cells harvested via LVF [9]. However, a previous study described CB as the most reliable method for diagnosing bovine cytological endometritis [10].

Mateus *et al*. [11] found that ultrasound uterine measurement is convenient and allows for reliable result comparison. Ultrasonographic intrauterine fluid determination 3 weeks postpartum exhibits good sensitivity and specificity and is reliable for diagnosing endometritis [8,10].

Most of the previous studies however were performed for endometritis in dairy cows, and only a few were conducted on beef cows. Endometritis incidence in beef cows in Malaysia is unknown and information in this regard is lacking. Thus, this study aimed to evaluate the ultrasound method and its agreement with the endometrial cytology method, which is used to diagnose the cytological endometritis in beef cows. Furthermore, in order to compare the sensitivity and specificity between the two methods.

## Materials and Methods

### Ethical approval

This study was approved by the Institutional Animal Care and Use Committee, Universiti Putra Malaysia (Ref. UPM/IACUC/AUP-R099/2015; 10 February 2016).

### Animals

The study was conducted during 20-35 days postpartum. A total of 53 clinically healthy beef cows (28 Brangus and 25 Kedah–Kelantan breeds) were obtained from three beef farms between October 2015 and September 2016. The farms are located in Serdang, Selangor (temperature, 28°C; relative humidity, approximately 70%). The cows were in the age range of 3-7 years and their body weights were from 300 to 450 kg. Moreover, the cows were managed under a free grazing system and supplemented consistently with feeds consisting of alfalfa, corn silage, beet pulp, cottonseed, soya bean, corn, and barley. The herd used numerous bulls for natural mating after a voluntary waiting period of approximately two months. The body condition scores of the cows were evaluated using a five-scale point as described by Dubuc *et al*. [3].

### Ultrasound examination

Ultrasound examination was conducted to determine the uterine cervix diameter and fluid accumulation in the uterine lumen [8,10,12]. All cows were scanned using B-mode ultrasound attached with a linear probe of 5MHz frequency (Sonosite VET 180 Plus, Bothell, WA, USA). The cows were grouped into two categories: Cytological endometritis and healthy cows. Cows with cervix diameter measurement (CM) higher than 5 cm and uterine horns containing fluid in the uterus (FIU), regardless of the amount or nature (hyperechogenic or hypoechogenic), upon ultrasonography, were classified under the endometritis group, as described by Meira *et al*. [12]. A cow was categorized as healthy when its uterine cervix diameter was <5 cm, with no abnormal discharge externally or in the uterus based on ultrasonographic findings, as described by Meira *et al*. [12].

### Endometrial cytology by CB method

**Figure-1:**
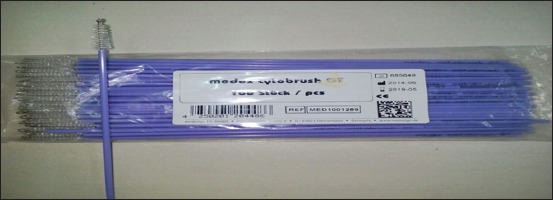
Image of the cytobrush used to get endometrial cytological samples.

**Figure-2:**
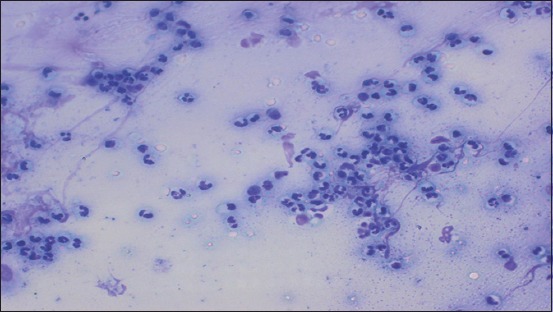
Cytological smear obtained by cytobrush method from subclinical endometritis cow stained with Giemsa stain, observed by light microscope 400 magnification. Slide shows high infilteration of PMN cells (neutrophils).

Endometrial cytological samples were collected using a sterile CB Plus GT (Figure-1) (Medscand Medical, Germany) modified for bovine use, as described by Madoz *et al*. [13]. The handle was shortened to 2 cm and threaded to a stainless steel rod (Artificial insemination gun; 65 cm length; 4 mm outside diameter). The CB and stainless steel rod attachments were guarded with a plastic sheath (Chemise Sanitaire, IMV Technologies, France) to avoid vaginal contamination, lubricated (Triad Sterile Lubricating Jelly, H&P Industries Inc., Mukwonago, WI, USA), and introduced into the vagina. Subsequently, a sleeved arm was introduced into the rectum to facilitate insertion of instruments through the genital tract and cervix. When the device passed the cervix, the CB was exposed and turned multiple times to obtain the cellular material from the adjacent endometrium. The CB was withdrawn from the genital tract, and the sample was rolled onto a glass slide. All slides were fixed with methanol for 30 min, stained with 5% Giemsa stain for 3 min, and dried. All slides were evaluated by counting 300 cells at 400× magnification (Leitz Laborlux-S, Wetzlar, Germany) to determine the % of neutrophils (%PMN) (Figure-2). Endometrial threshold values ≤8% were used, as described by Madoz *et al*. [13], to determine the occurrence of endometritis in the farms 20-35 days postpartum.

### Statistical analysis

All statistical analyses were performed using the SPSS software (version 18.0, IBM SPSS Inc., Chicago, USA) and Excel 2007. First, numerical data were tested using the Kolmogorov–Smirnov test for normality distribution. The endometritis occurrence was recorded from the clinical examination results of endometrial cytological samples. The agreement between endometrial cytological and ultrasound inspection findings was compared using kappa analyses. Differences were considered significant at p<0.05.

## Results

Results obtained using the CB method showed that six cows (6/53, 11.3%) and five cows (5/53, 9.4%) at 4 and 5 weeks postpartum showed PMN ≥ 8%, which is indicative of cytological endometritis.

Ultrasound evaluation 4 weeks postpartum showed that 18 cows had FIU (Table-1); this result presented a 0.29 kappa agreement with the cytological method, as well as 83.3% sensitivity and 73.3% specificity. At 5 weeks postpartum, 12 cows were FIU-positive, which translated to a 0.38 kappa agreement, 80% sensitivity, and 83.3% specificity (Tables-2 and 3). According to the CM 4 weeks postpartum, 13 cows showed more than 5 cm and yielded a 0.32 kappa agreement, 66.6% sensitivity, and 80.8% specificity. At week 5, cervical measurements revealed that seven cows were positive for endometritis, thereby giving a 0.43 kappa value, 60% sensitivity, and 91.7% specificity. When the parameters were combined (FIU + CM) to increase the accuracy of diagnosing endometritis, an improved kappa agreement was observed between ultrasound and CB methods. Consequently, a 0.67 kappa value, 66.6% sensitivity, and 91.5% specificity were obtained at week 4. Similarly, at 5 weeks postpartum, the same comparison resulted in a 0.49 kappa value, 60% sensitivity, and 93.8% specificity (Table-3).

**Table-1:** Agreement among diagnostic methods for endometritis 4 weeks postpartum.

Week 4 post calving	Cytobrush		Kappa (p=value)

	PSN≥8%	PMN<8%	
Ultrasound (FIU)			
Positive	5	13	K=0.29 (p=0.007)
Negative	1	34	
Ultrasound (CM)			
≥5	4	9	K=0.31 (p=0.01)
<5	2	38	
Ultrasound (FIU+CM)			
Positive	4	4	K=0.50 (p=0.00)
Negative	2	43	

Endometrial cytology results showed that beef cows with %PMN>8 are healthy, and those with≥8 are suffering from endometritis 4 weeks postpartum. FIU is the fluid in uterine, which is negative (no fluid) or positive (present fluid) upon ultrasonographic evaluation. CM is the uterine cervix diameter: >5 cm, healthy; ≥5 cm, endometritis, upon ultrasonographic evaluation. Kappa statistic measures the level of agreement between tests, where 1=complete agreement and 0=no agreement

**Table-2:** Agreement among diagnostic methods for endometritis 5 weeks postpartum.

Week 5 post calving	Cytobrush		Kappa (p=value)

	PMN≥8%	PMN<8%	
Ultrasound (FIU)			
Positive	4	8	K=0.38 (p=0.001)
Negative	1	40	
Ultrasound (CM)			
≥5	3	4	K=0.43 (p=0.001)
<5	2	44	
Ultrasound (FIU+CM)			
Positive	4	3	K=0.49 (p=0.00)
Negative	2	45	

Endometrial cytology results showed that beef cows with %PMN>8 are healthy, and those with≥8 are suffering from endometritis 5 weeks postpartum. FIU is the fluid in uterine, which is negative (no fluid) or positive (present fluid) upon ultrasonographic evaluation. CM is the uterine cervix diameter: >5 cm, healthy; ≥5 cm, endometritis, upon ultrasonographic evaluation. Kappa statistic measures the level of agreement between tests, where 1=complete agreement and 0=no agreement

**Table-3:** Comparison of diagnostic techniques to endometrial cytology.

Method	Week 4			Week 5		
	
	Positive/total	Specificity (%)	Sensitivity (%)	Positive/total	Specificity (%)	Sensitivity (%)
FIU	18/53	72.3	83.3	12/53	83.3	80
CM	13/53	81	66.6	7/53	91.7	60
FIU+CM	8/53	91.5	66.6	6/53	93.8	60

FIU=Fluid in uterine, CM=Cervix measurement

## Discussion

Endometritis is one of the most common problems causing severe economic losses in dairy cows. This study aimed to compare the different methods used for diagnosing endometritis in beef cows. The cytological endometritis occurrences were 11.3% and 9.4% at 4 and 5 weeks postpartum, respectively, as determined by the CB endometrial cytology as the standard method. The present cytological endometritis percentage agreed with that of a previous survey (11.8%) in dairy cows [10] but it is less than that of another study (17%) in beef cows [14]. In one of the previous studies in beef cows, 17% of Angus cows (2-78 days postpartum) tested positive for cytological endometritis, as assessed by low-volume fluid (LVF) method [14].

The present findings are in agreement with previous studies that showed a reduced mean of PMN and endometritis as the postpartum period approaches the completion of histological involution [13].

The low cytological endometritis occurrence in this study may be due to the decreased rate of uterine infections in beef cows compared with that of dairy cows, which are under more stress milking) than most of the beef cow herd. Moreover, beef cows exhibit a high pregnancy rate during breeding season with proper management; furthermore, uterine contaminations can be controlled, especially after the resumption of the ovarian cycle [14]. At present, no consensus exists between studies regarding the threshold value when diagnosing cytological endometritis, as well as the most suitable time for uterine sample collection. Many cutoff values of PMN% (within 5-18) are used to diagnose cytological endometritis through CB and LVF [13,15-17]. Kasmanickam *et al*. [8] depended on >18% PMNs as a threshold value for 20-33 days postpartum and >10% PMNs for 34 and 47 days postpartum. Furthermore, Gilbert *et al*. [2] used >5% PMNs as a significant cutoff point for diagnosing bovine cytological endometritis using lavage 40-60 days postpartum. Other studies depended on these %PMN thresholds according to the effects on reproductive performance [17,18]. The lower prevalence of cytological endometritis found in the present study compared with other studies elsewhere might be be due to different geographical and environmental factors. Another reason might be related to differences in the endometrial cells used to evaluate PMN% in the endometrial samples. For example, in this study, we counted 300 cells per slide, but some authors counted only 100 cells [10].

Obtaining the most ideal method to diagnose cytological endometritis accurately using high sensitivity and specificity is difficult [1]. Numerous studies have investigated factors, such as nutrition, metabolic disorders, uterine infections, and genetic factors, that affect the pregnancy rate in cow herds [19]; however, other studies used reproductive performance as an indicator to evaluate these diagnostic methods [4].

This study focused on the comparison between the ultrasound technique and the cytological method in diagnosing cytological endometritis in beef cows. At 4 and 5 weeks postpartum, the involution of the bovine genital tract was almost complete in healthy cows but delayed among infected cows. The cows with uterine cervix diameters higher than 5 cm after week 4 developed uterine diseases; moreover, these cows may exhibit reduced fertility in the future [4]. Delayed uterine involution and uterine contamination with bacterial species postpartum are associated with uterine fluid accumulation, which is detected by ultrasound examination [11]. Our study showed a weak agreement between ultrasound evaluation and the cytological technique, especially at week 4, and a moderate agreement 5 weeks postpartum. These results agreed with previous studies that reported weak agreements between ultrasound measurements of uterine fluids and CB methods in diagnosing cytological endometritis among dairy cows [10,12]. These studies explained that both forms of endometritis are diagnosed using these methods; moreover, these endometritis forms include one that is associated with the cellular influx of PMN and another associated with the fluid accumulation inside the uterine lumen; furthermore, a low PMN percentage was observed with decreased uterine clearness [9,10]. In our study, both uterine fluid and cervical diameter were useful for detecting affected cows. Results showed improved sensitivity, specificity, and kappa agreement with the cytological method, which is the standard procedure for diagnosing cytological endometritis. This result was verified when the two parameters were combined to diagnose endometritis; consequently, high sensitivity (60%), specificity (93.8%), and 0.50 kappa agreement were obtained. These results were in agreement with those of Barlund *et al*. [10] and Meira *et al*. [12], who found that the ultrasound technique is a good, non-invasive, useful, and practical method to estimate uterine fluid or cervical diameter for diagnosing endometritis. The efficiency of the ultrasound method in the diagnosis of endometritis can be increased when combined with uterine fluids. Such combination yielded a 50% sensitivity, 88% specificity, and a 39% kappa agreement.

## Conclusion

The percentage of beef cows that were positive to cytological endometritis was low (PMN ≤8%) at 4 and 5 weeks postpartum. This study showed that the ultrasound method is useful and practical for diagnosing endometritis 4 and 5 weeks postpartum, especially when combined with detection of intrauterine fluid accumulation and measurement of cervical diameter thickness. This combination resulted in a high sensitivity of 60%, high specificity of 93.8%, and a 0.50 kappa agreement.

## Authors’ Contributions

NS designed and performed the experiments, drafted the paper, and analyzed the data. NY supervised the project and revised the manuscript. Both authors read and approved the final manuscript.
